# Presenilin 2 Is the Predominant γ-Secretase in Microglia and Modulates Cytokine Release

**DOI:** 10.1371/journal.pone.0015743

**Published:** 2010-12-29

**Authors:** Suman Jayadev, Amanda Case, Alison J. Eastman, Huy Nguyen, Julia Pollak, Jesse C. Wiley, Thomas Möller, Richard S. Morrison, Gwenn A. Garden

**Affiliations:** 1 Department of Neurology, University of Washington, Seattle, Washington, United States of America; 2 Department of Neuroscience, University of Iowa, Iowa City, Iowa, United States of America; 3 Department of Neurobiology and Behavior, University of Washington, Seattle, Washington, United States of America; 4 Department of Comparative Medicine, University of Washington, Seattle, Washington, United States of America; 5 Department of Neurological Surgery, University of Washington, Seattle, Washington, United States of America; Boston University School of Medicine, United States of America

## Abstract

Presenilin 1 (PS1) and Presenilin 2 (PS2) are the enzymatic component of the γ-secretase complex that cleaves amyloid precursor protein (APP) to release amyloid beta (Aβ) peptide. PS deficiency in mice results in neuroinflammation and neurodegeneration in the absence of accumulated Aβ. We hypothesize that PS influences neuroinflammation through its γ-secretase action in CNS innate immune cells. We exposed primary murine microglia to a pharmacological γ-secretase inhibitor which resulted in exaggerated release of TNFα and IL-6 in response to lipopolysaccharide. To determine if this response was mediated by PS1, PS2 or both we used shRNA to knockdown each PS in a murine microglia cell line. Knockdown of PS1 did not lead to decreased γ-secretase activity while PS2 knockdown caused markedly decreased γ-secretase activity. Augmented proinflammatory cytokine release was observed after knockdown of PS2 but not PS1. Proinflammatory stimuli increased microglial PS2 gene transcription and protein *in vitro*. This is the first demonstration that PS2 regulates CNS innate immunity. Taken together, our findings suggest that PS2 is the predominant γ-secretase in microglia and modulates release of proinflammatory cytokines. We propose PS2 may participate in a negative feedback loop regulating inflammatory behavior in microglia.

## Introduction

The development of specific and effective therapies for neurodegenerative disease will benefit from a clear understanding of the underlying pathogenic mechanisms. Significant clues to pathogenesis have come from the discovery of causative mutations in familial forms of sporadic disease. Mutations in the genes encoding presenilin 1 (PS1) and presenilin 2 (PS2) cause early-onset familial Alzheimer disease (AD). However, despite extensive efforts since their identification, how PS mutations cause neurodegeneration remains unclear.

PS are intramembrane proteases that form the catalytic component of the γ-secretase complex. More than 50 putative γ-secretase substrates have been identified which regulate a variety of cellular mechanisms including inflammation, development, and synaptic plasticity [1]. Many PS mutations lead to decreased generation of PS cleavage products including amyloid-beta 40 (Aβ40) and the Notch intracellular domain [2], [3], [4], [5] demonstrating that disease associated PS mutations may result in loss of normal function. Premature truncation mutations in PS have been reported in early onset-AD cases, supporting haploinsufficiency or dominant negative actions as a possible pathogenic mechanism in the development of CNS disease [6], [7]. Conditional double PS knockout mice (PScDKO) further support the hypothesis that loss of PS function contributes to neurodegeneration. These mice develop behavioral and neuropathological features typical for AD, including synaptic loss, neuronal loss, and neuroinflammation [8], [9], [10]. Thus, loss of PS function may be an important contributor to the observed neuropathology.

One potential biological function that may be impacted by loss of normal PS function is neuroinflammation. Regulation of PS during inflammation both *in vivo* and *in vitro* has been previously reported [11], [12]. In addition, mutant PS expressing and PScDKO mice both show abnormal neuroinflammatory responses suggesting that PS may function to regulate the innate immune response in the CNS [9], [13]. Since those PScDKO mice lack PS1 only in forebrain neurons, the findings may relate to the loss of PS2 in other cell types. However, how PS influences neuroinflammation has yet to be elucidated. Recent studies have demonstrated that PS2 is co-regulated with NFκB and immune signaling molecules in the Toll-like receptor (TLR) system including TLR4 and Myd88 suggesting a specific role for PS2 in established innate inflammatory pathways [11], [12].

It is widely accepted that microglia and the innate immune system are participants in the process of neurodegeneration [14], [15], [16], [17], [18]. Microglia can be both protective and injurious, causing neuronal injury through release of inflammatory cytokines, neurotoxins and excitotoxins [19], [20], [21], [22], [23]. While the variety of pathogen-associated molecular pattern (PAMPs) and damage- associated molecular pattern (DAMPs) microglial activating signals continue to be characterized, it is clear that TLR pathways mediate important signals that regulate microglia behavior in CNS disease [16], [21], [24], [25]. TLR activation initiates intracellular signaling resulting in activation of transcription factors such as AP-1 and NFκB and subsequent induction of inflammatory cytokines [25], [26]. Elevated proinflammatory cytokine serum levels are associated with both the acquisition of neurodegeneration and progression of cognitive decline [27], [28].

We demonstrate here that γ-secretase inhibition and specific loss of PS2 expression results in an exaggerated cytokine response by microglia. In microglia, PS1 and PS2 demonstrate compensatory regulation where knockdown of one leads to upregulation of the other, but only PS2 depletion correlated with decreased γ-secretase function. Taken together these studies support the hypothesis that microglial PS2 functions in a manner distinct from PS1 to downregulate proinflammatory cytokine release and thus may act as a novel molecular modulator of microglia behavior. Abnormal activity of PS2 either through mutation, or secondary to changes in the aging brain may lead to unchecked proinflammatory behavior with subsequent neuronal injury contributing to neurodegeneration.

## Materials and Methods

### Mice

All animals were housed and all experiments conducted according to the University of Washington IUCAC guidelines and approved by University of Washington IUCAC, Protocol # 2856-01. C57Bl/6 and PS2 knockout mice, B6.129P-Psen2tm1Bdes/J, were purchased from Jackson Laboratories.

### Microglia cell culture

Cortical tissue from postnatal day 3 or 4 (P3 or P4) wild-type or PS2 KO mice was dissected and cut into small pieces (∼1 mm^3^). Tissue pieces were incubated with 0.125% trypsin at 37°C for 25 min, followed by 1 mg/ml soybean trypsin inhibitor. Cells were dissociated by trituration in Neurobasal™ (Gibco, Great Island, NY) media containing 10 mM HEPES and 100 µg/ml DNase I. Glial cells were cultured in DMEM containing 10% horse serum and 20% L929 conditioned medium and incubated at 37° and 5% C0_2_ for 9–14 days before microglia were detached from the astrocyte layer via gentle tapping of flasks.

BV-2 cells were cultured in DMEM with 10% FBS. BV2 cell-lines stably expressing PS1 or PS2 shRNA were cultured in DMEM, 10% FBS and 5 mg/ml puromycin selection antibiotic. Five PS1 and 5 PS2 shRNA constructs were purchased from Sigma-Aldrich and two different shRNA sequences resulted in PS1 or PS2 knockdown. PS1 shRNA sequences: TRCN0000054503: CCGGCAAGCATGTCATCATGCTCTTCTCGAGAAGAGCATGATGACATGCTTGTTTTTG and TRCN0000054504: CCGGCGTTACAGTAGCACTCCTAATCTCGAGATTAGGAGTGCTACTGTAACGTTTTTG PS2 shRNA sequences: TRCN0000030525:CGGGCAGGCTTACCTTATTGTGATCTCGAGATCACAATAAGGTAAGCCTGCTTTTT and TRCN0000030528:CCGGCCTCGTGGTACTCTACAAGTACTCGAGTACTTGTAGAGTACCACGAGGTTTTT. Stably expressing lines were created per manufacturer's protocol. Briefly, 5×10^4^ cells per 35 mm dish were infected with one of 5 PS1 or PS2 shRNA lentiviral constructs. Twenty-four hours later, lentiviral containing media was replaced with standard BV2 culture media for another 48 hours then replaced with 5 mg/ml puromycin in DMEM 10% FBS. Antibiotic resistant single cells were selected and expanded.

### Western blot analysis

Primary cells were plated at 3×10^5^ per 35 mm dish in DMEM with 10% horse serum overnight. BV2 and virally transduced clonal cell line microglia were plated at 10^5^ per 35 mm dish in DMEM with 10% FBS. Cells were lysed in a protein extraction buffer containing 50 mM Tris-HCl; 150 mM NaCl; 5 mM EDTA, pH 7.4; 1% Na deoxycolate; 0.1% SDS; 1% Triton X-100; 1 mM PMSF; 7 mg/ml pepstatin; 5 µg/ml aprotinin; 5 µg/ml leupeptin; and 160 mM sodium orthovanadate, separated by sodium dodecyl sulfate polyacrylamide gel electrophoresis (SDS-PAGE), transferred to PVDF membrane (Bio-Rad, Hercules CA). Membranes were exposed to primary anti-PS2 Loop antibody (1∶1000 Calbiochem, #529594, San Diego CA), anti-PS1 CTF loop antibody 3109 (1∶1000, kind gift of Dr. Jochen Walter) and mouse anti-βactin (1∶100,000, Vector Laboraties) overnight at 4°C, then exposed to appropriate secondary antibody (1∶1000 anti-mouse or 1∶1000 anti-rabbit HRP conjugated, Amersham, Pittsburgh, PA) for 1 hour at room temperature. Proteins were visualized with Enhanced Chemiluminescence (Pierce, Rockford, IL). Quantification of protein was performed employing Image J software (NIH). TACE expression was evaluated using goat anti-TACE (1∶500, Santa Cruz Biotechnology, #6416, Santa Cruz, CA) primary antibody and donkey anti-goat (1∶5000, # 2056, Santa Cruz biotechnology, Santa Cruz, CA) secondary.

### RT-PCR

Primary microglia were cultured for 24 hours, then stimulated with 10 u/ml IFNγ for 24 hours after which RNA was collected using the Roche RNA isolation kit (Roche Diagnostics, Indiana, IN). PSEN2 expression was measured by real-time-PCR using TaqMan Gene Expression Assay on Demand (Applied Biosystems, Carlsbad, CA), and analyzed with Step-One Plus software from ABI.

### Cytokine measurement

Primary microglia plated at 5×10^4^ cells per well in poly-D-lysine coated 96 well plates were cultured overnight followed by stimulation with vehicle, 10 u/ml IFNγ (R&D systems, Minneapolis MN), 100 ng/ml lipopolysaccharide (LPS) (catalog # L2654, strain 026:B6 *Escherichia coli*, Sigma-Aldrich, St. Louis, MO) or amyloid-β (1–42) (catalog #62-0-80, American Peptide, Sunnyvale, CA) for 24 hours. Amyloid-β 1–42 (Aβ42) was reconstituted in sterile water to a stock concentration of 350 µM and stored at −20 prior to use. Fibrillar Aβ42 was prepared as previously described [29], [30]. Aβ42 peptides were incubated at 37° for 3 days and used at a final concentration of 10 µM. BV2 and shRNA expressing clones were plated at 10^4^ per well and stimulated as above. Conditioned media was then collected and subjected to TNFα or Il-6 ELISA analysis per manufacturer's protocol (R&D systems). Multiplex cytokine analysis was performed on conditioned media collected from primary microglia and evaluated using Millipore bead array and analyzed via Luminex 100IS system (n = 3 separate experiments). To evaluate effect of γ-secretase inhibition cells were first exposed to the pharmacological γ-secretase inhibitor, N-[(3,5-Difluorophenyl)acetyl]-L-alanyl-2-phenyl]glycine-1,1-dimethylethyl ester (DAPT) for 12 hours prior addition of IFNγ or LPS for 24 hours.

### γ-secretase activity assay

Two constructs were employed to measure γ-secretase activity in primary and cell line microglia. The APP-Gal4-VP16 and Gal4-Luc-GFP constructs have been previously described [2] and were subcloned into the pSL6 lentiviral shuttle vector [31]. Primary microglia were transduced with lentivirus which results in high efficiency and without induction of microglia proinflammatory activation [32]. Lentivirus was prepared through the Fred Hutchinson lentiviral core facility (Seattle, WA, NIH Grant DK 56465). Primary and cell line microglia were plated at 10^4^ cells per well in 96 well plates. Four hours after plating, microglia were incubated with lentiviral γ-secretase assay constructs at MOI of 1-3 for 18 hours followed by replacement with fresh culture media. γ-secretase activity was determined by luciferase activity and measured 72 hours after initial infection. Cells were lysed and subjected to luciferase assay as recommended by manufacturer's protocol (Promega, Madison, WI).

## Results

### γ-secretase Inhibition Augments the Microglia Proinflammatory Response

**Figure 1:**
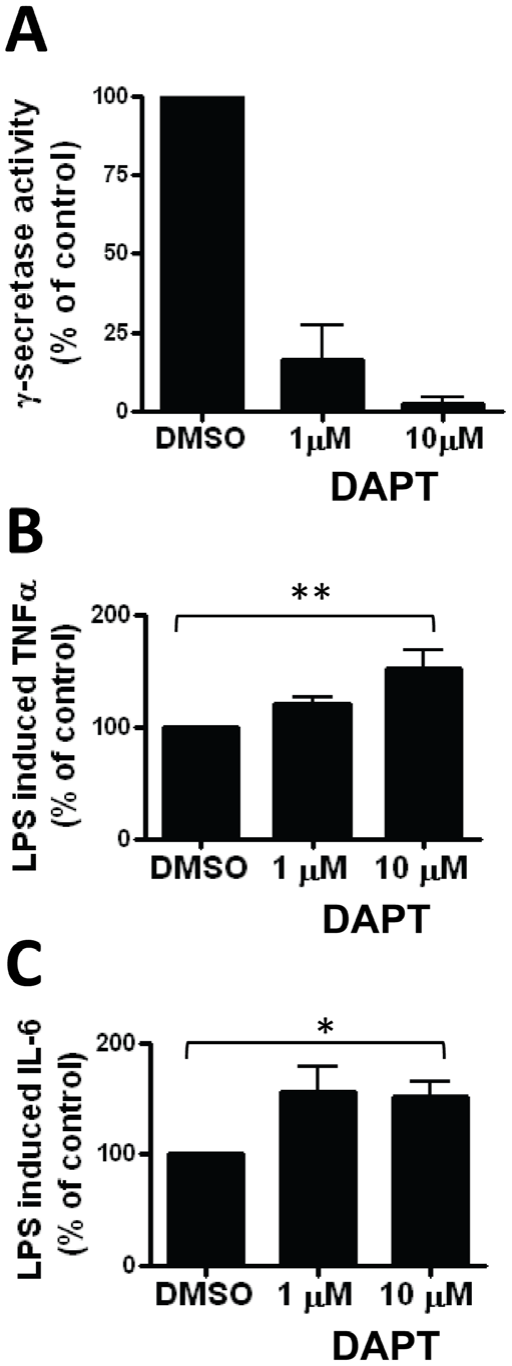
Pharmacological γ-secretase inhibition enhances proinflammatory cytokine release in primary microglia. Microglia were infected with luciferase reporter γ-secretase assay constructs and exposed to vehicle control (DMSO), 1 µM or 10 µM of the pharmacological γ-secretase inhibitor, DAPT. A) DAPT suppresses γ-secretase activity as measured by luciferase reporter assay. Data are means ±SEM of 3 independent experiments. Primary cultured microglia were pre-incubated with DMSO, 1 µM or 10 µM DAPT for 12 hours followed by addition of 100 ng/ml LPS. (B) TNFα and (C) IL-6 release were measured after LPS stimulation in DAPT or DMSO treated cultures. There is a significant effect of DAPT on TNFα (**p<0.01 by ANOVA) and IL-6 (*p<0.05 by ANOVA) release. Data represent the mean ± SEM of 3 independent experiments.

To study the role of the γ-secretase complex in CNS resident innate immune cells, we employed a pharmacological inhibitor to block γ-secretase activity in cultured primary murine microglia and assessed microglia proinflammatory responses. We first confirmed the presence of an intact γ-secretase complex that is repressible by the pharmacological γ-secretase inhibitor, DAPT. The γ-secretase activity luciferase reporter assay has been previously described and validated [2]. Briefly, γ-secretase mediated cleavage of an APP-Gal4VP16 fusion protein results in release of the intracellular C-terminal fragment (analogous to the C-terminal fragment of endogenous APP) that subsequently initiates transcription of a luciferase gene which is downstream of a promoter containing Gal4 binding sites. Cells transduced with the reporter constructs were exposed to DAPT or vehicle control (DMSO) for 24 hours followed by collection of lysate for luciferase assay. DAPT inhibits γ-secretase mediated cleavage of APP in a dose dependent manner (Fig. 1A).

In the diseased brain microglia can be stimulated by a variety of sterile and pathogenic stimuli. We chose to stimulate primary microglia with the canonical TLR4 stimulus, lipopolysaccharide (LPS) to examine the interaction between TLR stimulation and γ-secretase. We incubated primary microglia with DAPT or vehicle control (DMSO) for 12 hours prior to 24 hour stimulation with LPS then measured the release of TNFα and IL-6, two proinflammatory cytokines increased in neuroinflammatory and neurodegenerative processes [33] by ELISA. Incubation with DAPT resulted in an augmented cytokine response to LPS (Fig. 1B). The increase in TNFα release was dose dependent and statistically significant (p<0.01 by ANOVA) while the IL-6 response was maximal at both doses of DAPT tested (p<0.05 by ANOVA) (Fig.1C). Treatment with DAPT alone (in the absence of LPS) does not induce detectable cytokine release from microglia (data not shown) suggesting that γ-secretase inhibition modulates proinflammatory responses rather than independently inducing a non-specific noxious injury. Similar results were found using the γ-secretase inhibitors L-685,458 and Compound E. These findings suggested that γ-secretase enzymatic activity participates in the microglia proinflammatory response and that inhibition of the γ-secretase complex leads to an exaggerated proinflammatory state in microglia.

### Presenilin knockdown leads to compensatory increase of the other Presenilin

**Figure 2:**
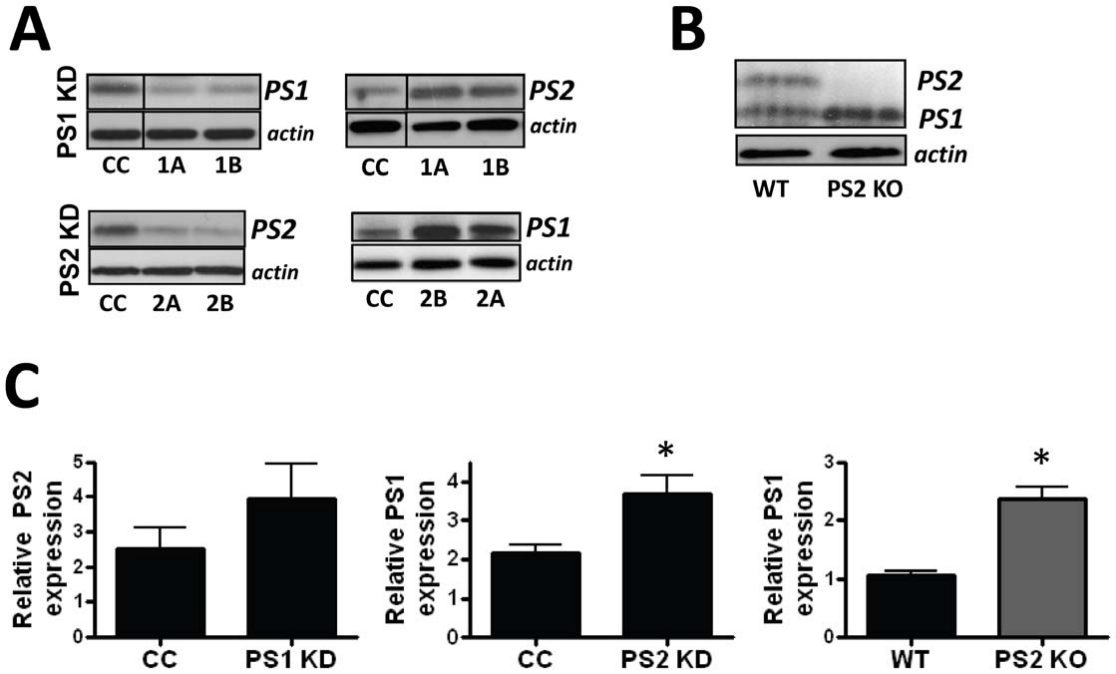
Decreased expression of one PS results in compensatory increase of the other PS. A) PS1 C-terminus migrating at ∼18 kDa and PS2 C-terminus migrating at ∼20 kDa are shown in representative western blot analyses of lysates prepared from non-target shRNA expressing control clone (CC) and 2 BV2 clonal cell lines (1A, 1B) stably expressing PS1 shRNA and 2 BV2 clonal cell lines (2A, 2B,) stably expressing PS2 shRNA. The panel of protein bands are taken from the same gel with irrelevant lanes removed from the figure. B) Western blot analysis of lysates prepared from primary microglia isolated from wild-type (WT) or PS2 knockout mice (PS2 KO). Antibodies to C-termini of PS1 and PS2 show protein bands at approximately 18 kDa and 20 kDa respectively. C) Densitometric analysis of Western blots measuring relative PS protein levels (normalized to actin) in PS knockdown cells and in PS2 KO cells. Data represent the mean ± SEM of 3 independent experiments (*p<0.05 by Student's t-test).

The γ-secretase complex is comprised of APH-1, nicastrin, Pen-2 and either Presenilin 1 or Presenilin 2 [34]. To further dissect the potential mechanism by which the γ-secretase complex influences cytokine release it is necessary to determine if one or both presenilins direct γ-secretase activity in the manner described above. Presenilins have overlapping, though distinct γ-secretase activity [35], [36]. The PS1 knockout mouse is embryonic and neonatal lethal with severe developmental defects. Therefore isolation of PS1 knockout primary microglia to study the impact of PS1 absence on microglia function is not practical. To circumvent the lack of an available source of PS1 knockout primary microglia, we used short hairpin loop RNAs (shRNA) to silence PS1 and PS2 in BV2 cells, a murine microglia cell line. Lentiviral constructs expressing shRNA against PS1 and PS2 and a puromycin resistance gene were employed to generate stable shRNA expressing BV-2 clones. To control for lentivirus infection and shRNA off-target effects, two unique shRNA sequences for each gene were used to generate multiple knock down clones. BV2 cells were also infected with a lentivirus expressing a non-target shRNA (control shRNA) against prokaryotic genes. Clonal cell lines were evaluated for PS1 and PS2 protein expression by Western blot analysis (Fig. 2A) and those lines expressing at least a 50% decline were employed for subsequent functional studies. There was no measurable effect on viability for any of the control, PS1 or PS2 knockdown cell lines. Previous work has suggested that PS1 and PS2 likely exist in a co-regulated balance [37]. We indeed found PS1 knockdown clones showed increased PS2 levels compared to non-target shRNA control lines while PS2 knockdown clones had mildly increased levels of PS1 compared to control lines (Figs. 2A and 2C). We found a similar compensatory increase of PS1 in the primary PS2 knockout microglia compared to controls (Figs. 2B and 2C). These findings demonstrate that in microglia, PS1 and PS2 expression levels are complementary to each other, such that reduction in one PS results in a compensatory increase of the other.

### PS2 is the predominant γ-secretase in microglia

**Figure 3:**
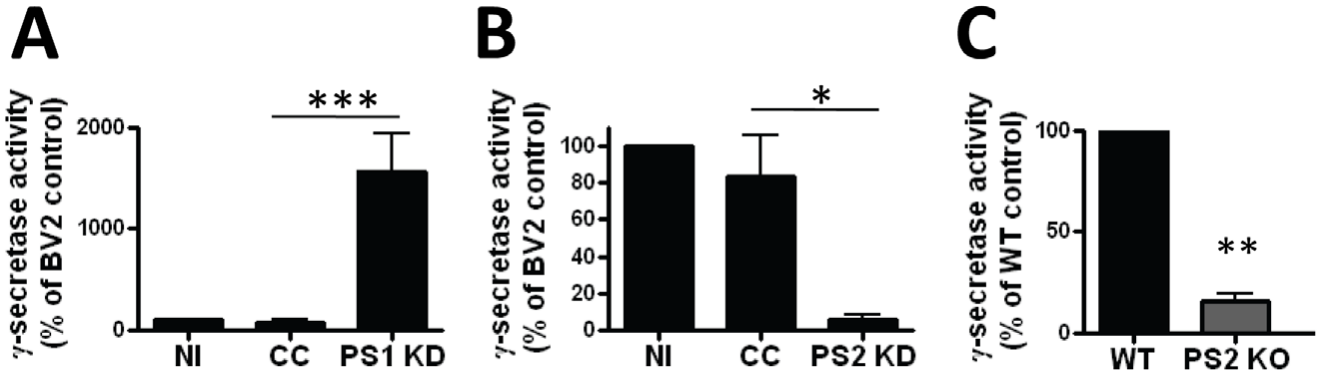
γ-secretase activity correlates with PS2, not PS1 protein expression. γ-secretase cleavage of an APP-Gal4 construct that induces luciferase expression was measured by determining luciferase activity in uninfected BV2 (NI), control non-target clone (CC) three PS2 shRNA expressing BV2 cell lines (PS2 KD) and two PS1 shRNA expressing BV2 lines. A) γ-secretase activity is approximately 10 fold increased in PS1 shRNA expressing BV2 clones. Data represent the mean ±SEM of 3 independent experiments (***p<0.001 by Tukey's post-test analysis). B) γ-secretase activity is significantly decreased in PS2 shRNA expressing BV2 clones Data represent the mean ±SEM of 3 independent experiments (*p<0.01 by Tukey's post-test analysis). C) γ-secretase activity as measured by APP-Gal4 cleavage induction of luciferase activity in WT and PS2 KO microglia demonstrating markedly impaired enzymatic activity in PS2KO cells. Data represent the mean ±SEM of 3 independent experiments (**p<0.005, by Student's t-test).

To determine the impact of PS knockdown on functional enzymatic activity we measured γ-secretase activity by the above described luciferase reporter assay [2]. Surprisingly, we found a disparate impact of specific PS knockdown on γ-secretase activity. PS1 knockdown led to dramatically increased γ-secretase activity (Fig. 3A). On the contrary, PS2 knockdown resulted in marked decrease in cleavage of APP in spite of elevated levels of PS1 (Fig. 3B). Similarly, primary PS2 KO microglia express more PS1 than wild type microglia, but have significantly impaired γ-secretase enzymatic activity compared to control (Fig. 3C). These novel findings indicate that while suppression of either PS leads to a compensatory increase in expression of the alternate PS, functional enzymatic activity of the γ-secretase complex as measured by APP cleavage in microglia is predominantly mediated by PS2.

### Deficiency of PS2 but not PS1 results in an augmented proinflammatory response

**Figure 4:**
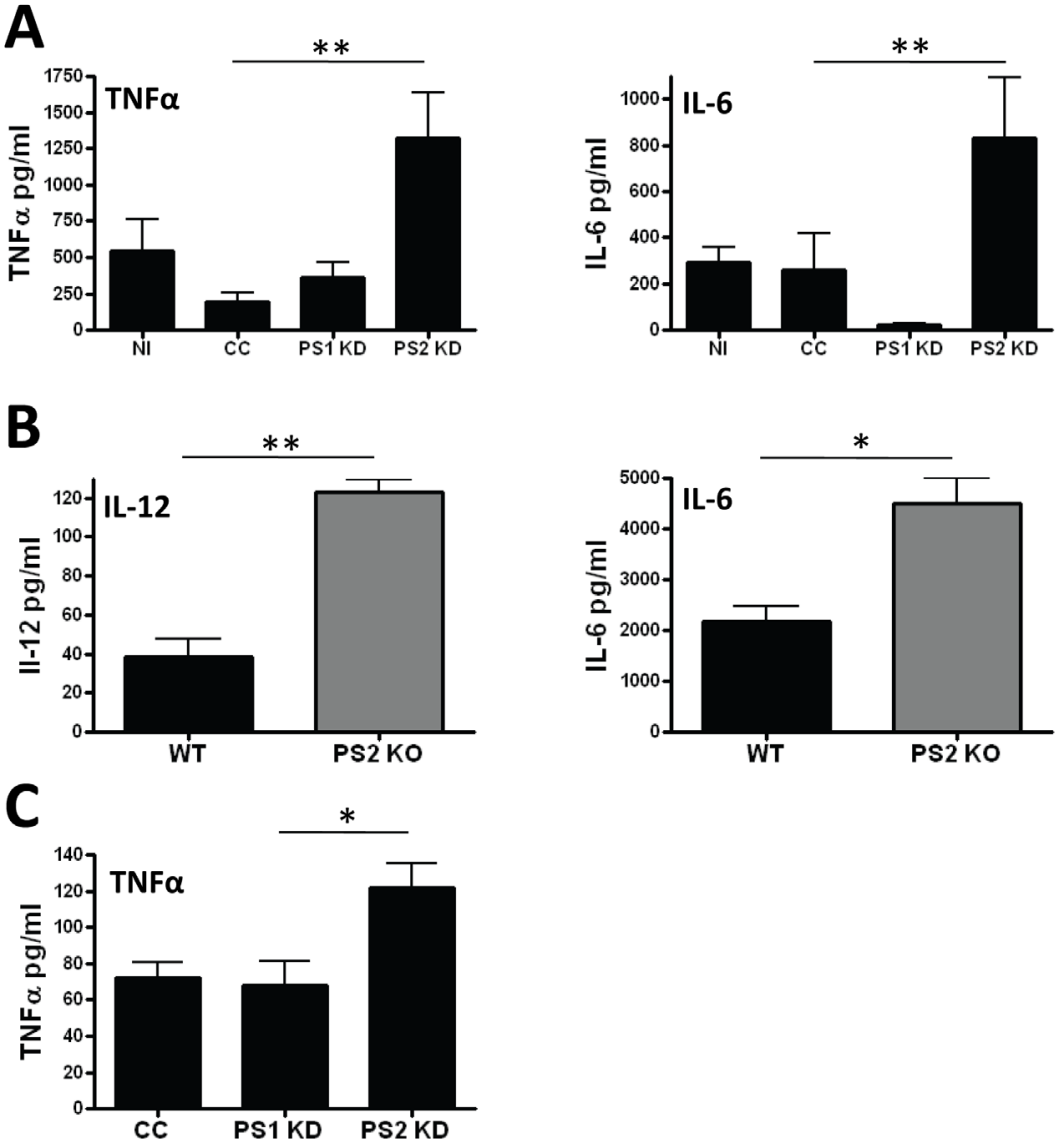
PS2 deficiency is associated with increased proinflammatory cytokine release. A) 100 ng/ml LPS stimulation results in significantly more TNFα and IL-6 release in three PS2 knockdown cell lines (PS2 KD) versus controls (CC). Data represent the mean ± SEM of 3 independent experiments (**p<0.01 by Tukey's post-test analysis). B) Cultured PS2 KO primary microglia stimulated with LPS for 24 hours released elevated levels of IL-12 and IL-6 compared to wild-type control cells as measured by multiplex cytokine array. Data represent the mean ± SEM of 3 independent experiments. (*p<0.05, **p<0.01 by Student's t-test). C) Twenty-four hour stimulation with 10 µM Aβ42 results in significantly increased TNFα release in PS2 KD cells versus non-target control (CC) Data represent the mean ± SEM from 3 independent experiments (*p<0.05).

We determined that γ-secretase inhibition leads to increased proinflammatory cytokine release in microglia and that PS2 is the primary mediator of microglial γ-secretase activity. We therefore expected that PS2 knockdown microglia may demonstrate a similar augmentation of proinflammatory cytokines as was observed in response to pharmacological γ-secretase inhibition. Uninfected BV2 cells, control shRNA infected clones and PS1 and PS2 knockdown cell lines were compared for their response to the proinflammatory stimulus, LPS for 24 hours. As would be expected if PS2 was the more functionally relevant PS in microglia, PS2 knockdown cell lines secreted more TNFα and IL-6 into conditioned media compared to control shRNA infected BV2 clones, uninfected BV2 controls and PS1 knockdown cell lines (Fig. 4A). To account for clone specific effects, three unique clones expressing one of two different PS2 shRNA sequences were averaged together for analysis of PS2 knockdown and two unique clones expressing two different PS1 shRNA sequences were averaged together for analysis of PS1 knockdown. Three independently derived non-target control shRNA clones were averaged together for analysis. At baseline, none of the knockdown cell lines or uninfected BV2 cells release proinflammatory cytokines above the limit of detection (data not shown). The effect of PS2 knockdown on TNFα and IL-6 response release after stimulation with LPS was significant (respectively, p<0.005 and p<0.02 by ANOVA). In contrast, there was no significant effect of PS1 knockdown on the TNFα response to LPS (Fig. 4A) despite the marked reduction in PS1 protein. Interestingly, IL-6 release was markedly reduced in PS1 knockdown cells and t-test analysis revealed a statistically significant decrease in IL-6 in PS1 knockdown cells compared to naïve BV2 cells (p<0.02).

Since TNFα release can be induced by increased cleavage of its membrane associated precursor, we addressed the possibility that PS2 might regulate TNFα release by reducing the expression of the TNFα converting enzyme (TACE). We evaluated the effect of PS2 knockdown on TACE expressing using Western blot analysis and observed no significant effect of PS2 knockdown on TACE expression (Fig. S1). This suggests that PS2 does not alter TNFα release by impacting TACE protein levels. Future studies are aimed at measuring TACE activity in the context of PS2 knockdown and knockout.

PS2KO primary microglia also demonstrated reduced γ-secretase activity, suggesting that if γ-secretase activity modulates the pro-inflammatory response, then PS2 knockout microglia would behave like the PS2 knockdown BV2 cell clones and DAPT treated microglia. As predicted, PS2KO primary microglia demonstrated increased proinflammatory cytokine release. We observed that PS2 KO microglia released significantly increased levels of IL-6 (Fig. 4B). To determine if IL-6 is an isolated cytokine regulated by PS2 expression in primary cells we performed multiplex cytokine analysis on conditioned media from wildtype and PS2 KO microglia. We found that in addition to IL-6, IL-12 levels were increased after 24 hours LPS stimulation in PS2 KO cells compared to wildtype. PS2KO microglia did not release significantly increased TNFα compared to wildtype microglia as had been observed with PS2 knockdown (Table S1). To determine if IL-12 is also increased in PS2 KD cells we assayed IL-12 levels in BV2 cells after LPS stimulation. We did not detect any measurable IL-12 in BV2 cells or BV2 cell line clones. In total, PS2 deficiency impacts multiple proinflammatory cytokines as measured in our assay. Taken together these results further suggest that PS2 likely regulates a general pattern of proinflammatory cytokine response.

To determine whether an AD relevant stimulus would reveal a similar augmentation of TNFα by PS2 knockdown, we employed 10 µM Aβ42 to activate microglia. While LPS alone can induce measurable cytokine release in BV2 cells, Aβ42 stimulation required priming by IFNγ, an established component of classical activation of monocytes [38], [39]. Stimulation with Aβ42 alone did not induce cytokine release and incubation with IFNγ did not result in a significant difference in TNFα release between PS1 knockdown, PS2 knockdown and non-target clones (data not shown). IFNγ primed PS2 knockdown cells released significantly higher levels of TNFα compared to primed non-target controls (p<0.05 by ANOVA) after 24 hours stimulation with Aβ42 (Fig. 4C). These data suggest that PS2 participates in the regulation of the microglia response to LPS, a canonical TLR4 ligand as well as to Aβ42 a molecule known to play a role in neurodegenerative disease.

### Proinflammatory Stimuli Increase Presenilin-2 Protein in Cultured Microglia

**Figure 5:**
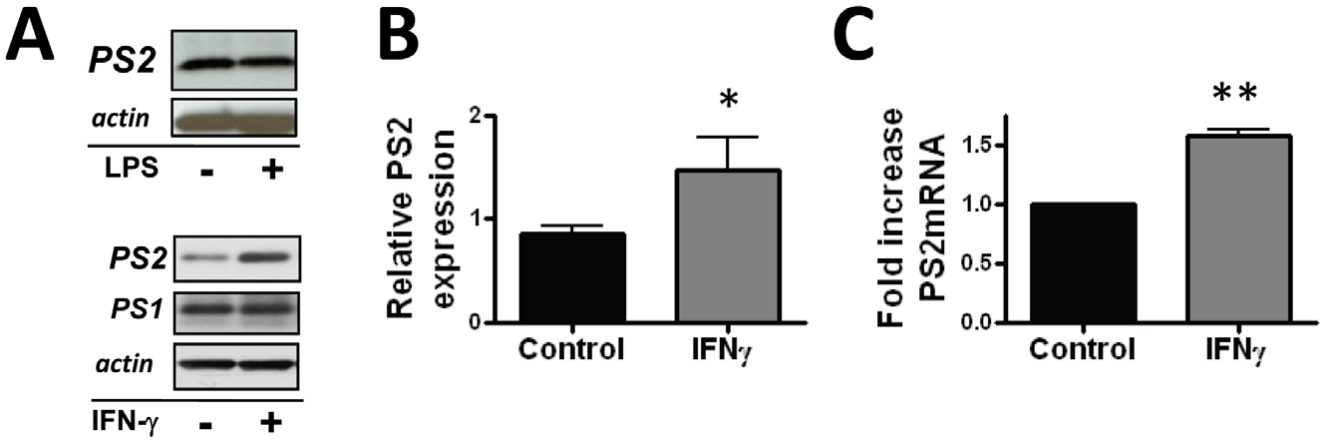
PS2 protein is increased in microglia activated by IFNγ. A) Western blot analysis demonstrating the PS2 C-terminus migrating at ∼20 kDa. Twenty-four hour stimulation with 10 u/ml IFNγ induces increased levels of PS2 expression in lysates prepared from primary microglia, while 100 ng/ml LPS does not induce PS2 expression. B) Quantitative densitometry on Western blots of lysates from 4 independent cultures shows significantly increased induction of PS2 by IFNγ. Data represent the mean ± SEM of 4 independent experiments (*p<0.05; Student's t-test). C) Twenty-four hour stimulation of primary microglia with IFNγ results in significantly increased PS2 mRNA. Data represent the mean ± SEM of 3 independent experiments (** p<0.01 by Student's t-test).

Our findings suggest that PS2 acts to downregulate the proinflammatory response in microglia as PS2 deficiency is associated with a heightened release of proinflammatory cytokines in response to LPS stimulation. Whether PS2 expression itself could be regulated by proinflammatory stimuli remained unknown. We hypothesized that PS2 participates in a negative feedback loop in the proinflammatory response, and therefore its expression in microglia may be modulated by LPS induced stimulation. However, we observed that PS2 expression was not altered in primary microglia stimulated with LPS for 24 hours (Fig. 5A). While TLR4 signaling is an important component to the innate immune response in CNS disease pathogenesis, it is one of multiple inflammatory pathways mediating the microglial response to damage and pathogen stimuli. Interferon-γ (IFNγ), a mononuclear cell regulatory cytokine proposed to play a role in neurodegeneration, induces monocytes to adopt an activation state characterized by release of proinflammatory cytokines and cytotoxic molecules [38], [40]. Mononuclear cells isolated from AD patients secrete higher levels of IFNγ compared to controls [41] and IFNγ mediates a diverse set of inflammatory responses in animal models of neurodegeneration and neuroinflammation [42], [43], [44], [45]. Therefore we evaluated the impact of IFNγ stimulation of microglia on PS2 expression. Western blot analysis of lysates prepared from cultured primary microglia stimulated with IFNγ or control for 24 hours demonstrated statistically significant increased PS2, but not PS1 expression (Fig. 5A–B). Regulation of PS2 protein levels may be transcriptional or posttranslational; therefore we measured PS2 gene expression via real-time PCR assay. We found that IFNγ induces increased PS2 transcript, demonstrating that increased PS2 expression, at least in part, results from induction of gene expression. These data suggest that while PS2 appears to interact with TLR4 mediated pathways in microglia its expression is sensitive to inflammatory signaling by IFN-γ. We hypothesize therefore that dysregulated proinflammatory signals during neurodegeneration may concurrently contribute to neurotoxicity while also specifically inducing PS2 expression as a counter-regulatory mechanism to dampen the proinflammatory response.

## Discussion

Here we describe a novel functional role for PS2 in the modulation of microglia behavior. Our study identifies PS2 as the predominant γ-secretase in microglia, an observation not previously reported. Our findings also support the hypothesis that PS2 (via its function as a γ-secretase) is a molecular modulator of microglia activation by acting as a repressor of proinflammatory responses. Furthermore, we present evidence that PS2 expression can be regulated by inflammatory stimuli. Therefore PS2 could participate in a negative feedback loop whereby increased expression of PS2 by inflammatory stimuli leads to increased PS2 mediated γ-secretase activity and subsequent dampening of the proinflammatory response.

In the healthy CNS microglia constantly monitor their local environment. During neurodegeneration the loss of factors that normally repress microglia may initiate microglial “activation” behaviors [21]. How microglia initiate these behaviors in response to environmental signals has not been elucidated clearly. Studies have begun to identify both extracellular signals and signal transduction pathways that lead to the diversity of microglial behavior, although there are likely many regulators of microglial function yet to be identified. We have discovered that PS2 is a mediator of microglia proinflammatory cytokine release.

There are several potential mechanisms through which PS2 may act in microglia. γ-secretase substrates include numerous proteins known to regulate innate immune responses including notch, low-density lipoprotein receptor-related protein 1 (LRP1), interleukin-1 (IL-1) receptor type 1, IL-1 R2, CX3CL1, CXCL16, and the MHC class I protein, HLA –A2 [46], [47], [48], [49], [50], [51]. The notch family of proteins are γ-secretase substrates and well known transcriptional regulators [52] that influence the innate immune system [53], [54], [55]. Downregulation of notch signaling in microglia leads to enhanced levels of proinflammatory cytokines [56]. Macrophage activation induces notch signaling through its transcriptional target, Hes-1, to act as a negative feedback inhibitor of IL-6 transcription [55]. However, the regulation of γ-secretase activity in these studies was not the focus of investigation nor is it clear whether PS1 or PS2 was acting as the predominant γ-secretase in those cell types. These reports are consistent with our finding that interference with γ-secretase activity either pharmacologically or through decreased PS2 expression leads to an exaggerated proinflammatory response. Based upon our data, it is possible that PS2 specific enzymatic activity may be the upstream regulator of the notch pathway in microglia. There is also evidence that PS modulates cytokine release in immune cells independent of notch transcriptional signaling pathways, though these pathways have not been evaluated in microglia [57]. Thus, while notch is a candidate mediator of microglia regulation by PS2, it is only one of several possible substrates.

We have shown that PS2 not only modifies the innate immune behavior of microglia but that PS2 is in turn regulated by the innate immune response. The PS2 promoter has potential binding sites for the transcription factors AP-1, NFκB and hypoxia-inducible factor-1 (HIF-1), all of which are induced in response to CNS injury or inflammation [58], [59], [60]. PS2 mRNA is significantly upregulated in human neural cell lines after exposure to inflammatory cytokines [61]. Further studies are needed to determine the mechanisms by which inflammatory stimuli lead to increased microglial PS2 protein expression. What role modulation of PS2 expression may have on the proteolytic cleavage of other γ-secretase substrates was not specifically addressed in these studies. Future experiments are aimed at identifying additional substrates for PS2 mediated cleavage in microglia.

In addition to demonstrating that PS2 expression in microglia is regulated by inflammatory signals, we have discovered that PS expression in microglial cells show compensatory regulation, where PS1 knockdown leads to increased PS2 and vice versa. Since germline absence of PS2 also leads to increased expression of PS1 in primary microglia cells, the observed compensation is unlikely to be secondary to the use of a transformed microglia cell line or an off-target effect of shRNA. This compensation occurs in a bi-directional fashion even though only PS2 knockdown or knockout results in decreased γ-secretase activity in microglia. Our finding that γ-secretase enzymatic activity correlates specifically with the level of PS2 expression suggests the novel hypothesis that PS2 is the predominant functioning γ-secretase in microglia. It is known that PS1 and PS2 have distinct expression patterns [62] and thus we speculate that the two proteins may possess distinct functions. It is possible that the two presenilins mediate distinct cell type specific functions in the CNS. PS2 may play the predominant role in regulating microglia while PS1 may have an analogous or distinct function in other cell types.

In patients with PS1 mutations, abnormal PS1 may still impact microglia function. Expression of mutant PS1 in a knock-in mouse model results in accentuated inflammatory behavior in microglia [13] and conditioned media from PS1 mutant expressing microglia negatively impact hippocampal neurogenesis [63]. However, it is possible that proinflammatory consequences of PS1 mutation may also be secondary to PS2 dysfunction [13] because studies have demonstrated that mutant PS1 can interfere with PS2 function or alter the dynamics of PS2 γ-secretase complex assembly [64], [65].

We observed that the impact of PS2 deficiency on microglia cytokine release was more dramatic than the pharmacological inhibition of γ-secretase activity. The role that PS2 γ-secretase independent mechanisms may influence microglia behavior was not specifically addressed in this study. It is known that PS2 has non-canonical (γ-secretase independent) mechanisms of action [1], [66], [67]. It is possible that γ-secretase independent functions specific to PS2 are mediated by interactions with unique binding partners not shared with PS1, such as the PDGF receptor [64]. PS2 deficiency may also have secondary impacts on microglia activation by influencing the regulation of intracellular calcium [68], [69], [70]. We observed slight differences in the repertoire of upregulated cytokines in PS2 knockdown versus PS2 KO microglia. There are multiple biological reasons that may potentially explain these observations. First, primary microglia are a heterogeneous population whereas the BV2 cell line is likely to behave more uniformly in response to stimuli. Second, because the PS2 KO cells are germline knockout and are completely deficient in PS2 compared to partial knockdown of PS2 in cell lines, the two cell types are not complete parallels. In aggregate, however, our data show that γ-secretase inhibition and PS2 deficiency induce an exaggerated proinflammatory response in both cultured microglia paradigms. Future studies are needed to investigate whether PS2 may also modulate microglia behavior via one of its non-canonical functions.

Our findings have potentially significant implications to current pharmacological therapeutic efforts using γ-secretase inhibitors (GSI) or modulators. We have demonstrated that γ-secretase inhibition in microglia leads to exaggerated proinflammatory cytokine release which may be detrimental to the local CNS environment. While these studies were not performed *in vivo*, they do underscore the potential impact of GSI's of the CNS innate immune system. Therapeutics designed to reduce Aβ deposition through inhibition of γ-secretase may paradoxically result in further neuronal injury secondary to an exaggerated proinflammatory CNS milieu. Additionally, while evaluating the efficacy and safety of γ-secretase inhibition and modulation for AD and other diseases it may be beneficial to be aware of potentially adverse impacts on the central and systemic immune system.

In summary, we report that PS2 is the predominant γ-secretase in microglia and that PS2 deficiency leads to an exaggerated proinflammatory microglial phenotype. Microglia have increased PS2 protein in response to proinflammatory stimuli *in vitro*, suggesting that PS2 expression may be upregulated by inflammatory stimuli as part of a negative feedback loop to downregulate microglial inflammatory processes. Further studies are underway to identify the mechanisms by which PS2 regulates microglia behavior as well as to determine whether PS1 and PS2 may mediate distinct roles in other neural cells or inflammatory cells outside the CNS. For patients with neurodegenerative diseases exacerbated by neuroinflammation, there are currently no available disease modifying therapies. Thus, further elucidation of PS2 function in microglia may provide novel and specific targets for therapeutic intervention for diseases of the CNS.

## Supporting Information

Figure S1 **TACE expression is not impacted by PS2 knockdown.** Lysates were prepared from non-infected BV2 cells (NI), non-target expressing control cell lines (CC), PS2 shRNA expressing cell lines and primary microglia, were separated by electrophoresis and transferred to PVDF membrane which was probed for TACE protein seen at approximately 85 kDa. There was no difference in TACE expression between BV2, non-target cells and PS2 shRNA expressing cells.
(TIF)

Table S1 **Multiplex analysis results.** Multiplex analysis of conditioned media from primary wildtype and PS2 KO microglia stimulated with LPS for 24 hours. Values shown are the result of 3 independent experiments. Wildtype and PS2 KO microglia release KC, Rantes and TNFα in addition to IL-6 and IL-12.
(DOCX)
