# Supplementary figures and images for: Presenilin 2 Is the Predominant γ-Secretase in Microglia and Modulates Cytokine Release
Source: PLoS One. 2010 Dec 29;5(12):e15743. doi: 10.1371/journal.pone.0015743 (PMC3012089; doi:10.1371/journal.pone.0015743)

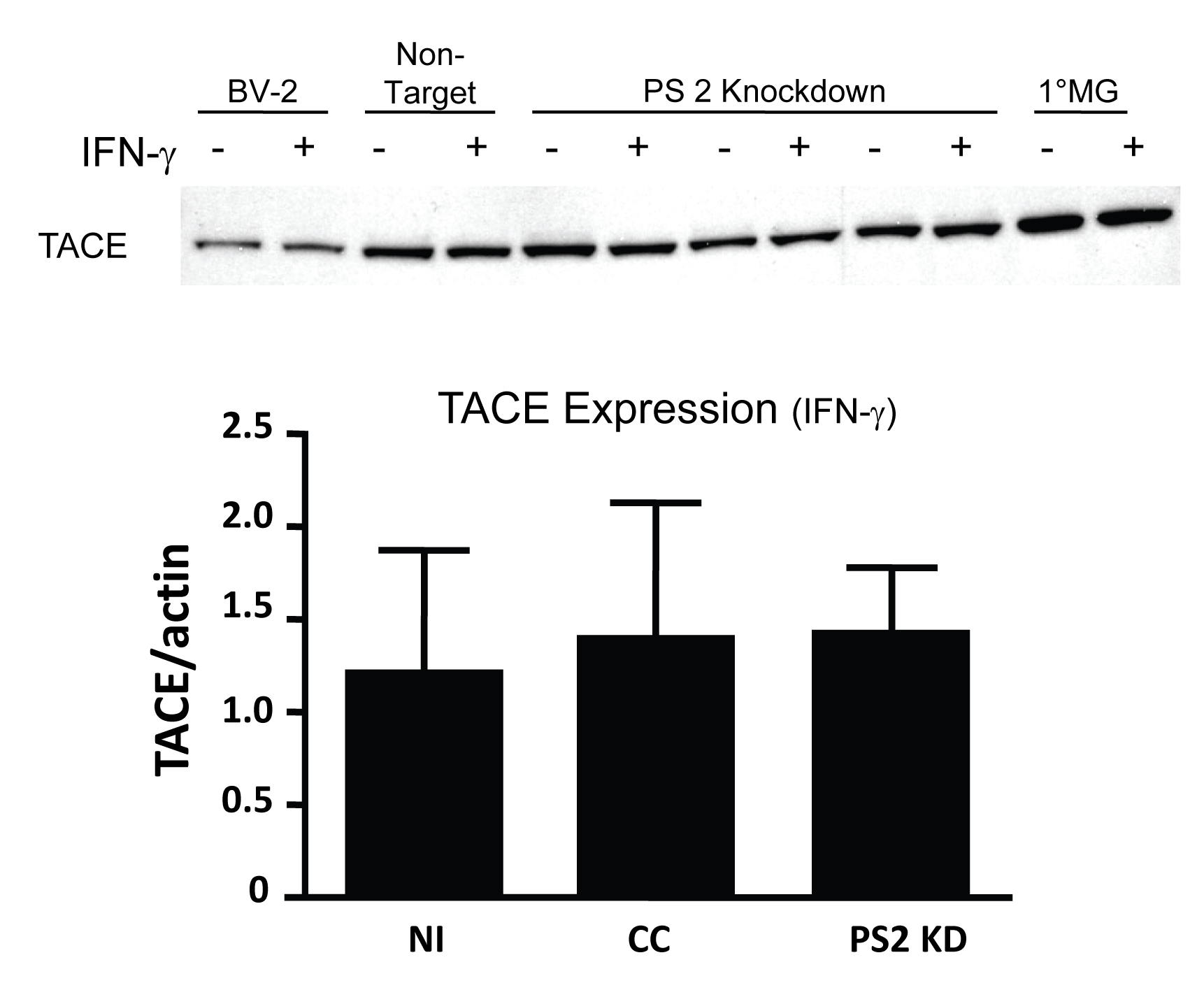

Supplement: Figure S1 — TACE expression is not impacted by PS2 knockdown. Lysates were prepared from non-infected BV2 cells (NI), non-target expressing control cell lines (CC), PS2 shRNA expressing cell lines and primary microglia, were separated by electrophoresis and transferred to PVDF membrane which was probed for TACE protein seen at approximately 85 kDa. There was no difference in TACE expression between BV2, non-target cells and PS2 shRNA expressing cells. (TIF) [file pone.0015743.s001.tif]
